# Predominance of Ancestral Lineages of *Mycobacterium tuberculosis* in India

**DOI:** 10.3201/eid1209.050017

**Published:** 2006-09

**Authors:** M. Cristina Gutierrez, Niyaz Ahmed, Eve Willery, Sujatha Narayanan, Seyed E. Hasnain, Devendra S. Chauhan, Vishwa M. Katoch, Véronique Vincent, Camille Locht, Philip Supply

**Affiliations:** * Institut Pasteur, Paris, France;; † Center for DNA Fingerprinting and Diagnostics, Hyderabad, India;; ‡ Institut Pasteur de Lille, Lille, France;; § Tuberculosis Research Center, Chennai, India;; ¶ National Jalma Institute for Leprosy and Other Mycobacterial Diseases, Agra, India

**Keywords:** tuberculosis, Mycobacterium tuberculosis, VNTR, spoligotyping, single nucleotide polymorphism, molecular epidemiology

## Abstract

Although India has the highest prevalence of tuberculosis (TB) worldwide, the genetic diversity of *Mycobacterium tuberculosis* in India is largely unknown. A collection of 91 isolates originating from 12 different regions spread across the country were analyzed by genotyping using 21 loci with variable-number tandem repeats (VNTRs), by spoligotyping, by principal genetic grouping (PGG), and by deletion analysis of *M. tuberculosis*–specific deletion region 1. The isolates showed highly diverse VNTR genotypes. Nevertheless, highly congruent groupings identified by using the 4 independent sets of markers permitted a clear definition of 3 prevalent PGG1 lineages, which corresponded to the "ancestral" East African–Indian, the Delhi, and the Beijing/W genogroups. A few isolates from PGG2 lineages and a single representative of the presumably most recent PGG3 were identified. These observations suggest a predominance of ancestral *M. tuberculosis* genotypes in the Indian subcontinent, which supports the hypothesis that India is an ancient endemic focus of TB.

## Abstract 2

Molecular epidemiologic findings suggest an ancient focus of TB.

Tuberculosis (TB) in humans has been described since ancient times. *Mycobacterium tuberculosis*, its main causative agent, is widely disseminated and is one of the most successful human pathogens today, with 2 billion persons infected. Most of the disease's effects are now concentrated in countries with few resources; India has the highest number of cases (*1*).

Because of the clonal structure of *M. tuberculosis* (*2**–**4*), comparative genotypic analyses from widespread geographic areas, such as the Indian subcontinent, or from different human populations can give unique insights into dissemination dynamics and evolutionary genetics of the pathogen (*5**,**6*). IS*6110* restriction fragment length polymorphism–based fingerprinting (*7*) has been used to study the mycobacterial population structure from southern India, northern India, and the Delhi region (*8**–**11*). However, IS*6110* fingerprinting is of limited use because a high proportion of *M. tuberculosis* strains have low copy numbers or are devoid of IS*6110* in several regions of India (*8**,**10*). IS*6110* typing also has a relative lack of portability, which hinders comparison between separate studies (*12*). Fingerprinting methods targeting polymorphic spacer sequences in the direct repeat (DR) region, including spoligotyping, have been used in some of these regions and in Bombay (*13**–**15*). However, when used alone, these methods considerably underestimate the clonal diversity (*16*). Because of these limitations, knowledge about the mycobacterial population structure in India remains incomplete.

More recently, molecular typing methods based on variable number tandem repeats (VNTRs) of genetic elements named mycobacterial interspersed repetitive units (MIRUs) (*17*) have been developed (*18**,**19*). MIRU-VNTR typing shows a discriminatory power close to that of IS*6110* fingerprinting and is particularly efficient in distinguishing *M. tuberculosis* isolates with few IS*6110* elements or none (*19**–**21*). MIRU-VNTRs are sufficiently stable to track epidemic strains (*19**,**20**,**22*).

We analyzed *M. tuberculosis* strain diversity in a sample of 91 isolates from 12 different regions, including northern, central, and southern India, by using a set of 21 VNTR loci, including the 12 MIRU-VNTR loci described previously (*17**,**18*) and 9 additional loci containing VNTRs of other interspersed genetic elements (*23**–**25*). All of these loci are collectively designated MIRU-VNTR loci in this study. Spoligotyping was used as a complementary technique because this procedure, albeit less discriminatory, is useful in identifying genotype families (*16**,**26**,**27*). In addition, single nucleotide polymorphism (SNP) genotyping on the *katG* and *gyrA* genes and genomic deletion analysis with *M. tuberculosis*–specific deletion region 1 (TbD1) were used to assess consistency of the genetic relationships obtained by VNTR typing and spoligotyping at a broader evolutionary level. SNPs in the *katG* and *gyrA* genes classify *M. tuberculosis* isolates into 3 principal genetic groups (PGGs) thought to have evolved sequentially from group 1 to group 3 (*2*). TbD1 is specifically present in a subset of PGG1 strains, but absent in other strains of PGG1, and in PGG2 and PGG3 strains; TbD1+ strains have therefore been proposed to constitute an ancestral lineage of *M. tuberculosis* (*28*). Using a combination of all 4 markers, we found that ancestral lineages prevail in our collection, which suggests an ancient focus of TB in the Indian subcontinent.

## Materials and Methods

### Strains and Genomic DNA Extraction

A sample of 100 clinical isolates of *M. tuberculosis* was initially selected; the isolates originated in 12 different regions, from northern, central, and southern India and part of eastern India (Table 1). For 9 isolates, mixed infections or laboratory cross-contamination was suspected after MIRU-VNTR typing (see Results), and they were excluded from further analysis. The isolates were collected from patients with pulmonary TB who had voluntarily visited their nearest medical college or hospital for diagnosis and treatment. Therefore, in most of the cases, the patients lived near the respective cities reported in Table 1. Patients were adults, 20 to 45 years of age, and represented both men and women, except those from Ranchi where all reported cases were in male army personnel. Information regarding the extent of disease and treatment status (new or recurrent cases of disease) was not available. From these hospitals, most of the isolates (designated hereafter as ICC, VA, VK, HA, BC, and ASN) were transported to the repository collection maintained at the Jalma Institute in Agra for further characterization and drug susceptibility testing, which was performed by the proportion method. Table 2 includes sensitivity profiles of isolates tested at the Jalma laboratory. The isolates from New Delhi were collected between March 1997 and March 1998, from Ahmedabad between November 1996 and July 1997, from Ranchi between February and March 1999, from Chandigarh in September 1997, from Bangalore in November 1996, from Jammu between May and July 2001, from Jaipur in May 2001, from Agra in May 2001, and from Varanasi between June and November 1999. The isolates designated as MHRC were from pulmonary TB patients who sought treatment at the Mahavir Hospital, Hyderabad, between 2000 and 2002. The isolates designated as TRC were from pulmonary TB patients at the Tuberculosis Research Centre, Chennai. *M. tuberculosis* DNA was extracted by using the standardized protocol as described (*7*).

**Table 1:** Origin of the *Mybacterium tuberculosis* isolates genotyped in this study

Origin	No. isolates*
Agra	2
Ahmedabad	7
Bangalore	6
Chandigarh	9
Chennai	9
Haridwar	2
Hyderabad	10
Jaipur	5
Jammu	6
New Delhi	26
North India	1
Ranchi	5
Shimla	1
Varanasi	2

*Nine isolates, originally selected and for which mixed infection or laboratory cross-contamination was suspected after typing with mycobacterial interspersed repetitive unit–variable-number tandem repeats (see Results), are not included.

**Table 2:** Available drug susceptibility profiles to rifampin (Rif) and isoniazid (Inh) of *Mycobacterium tuberculosis* isolates

Isolate no.	Origin	Sensitivity to Rif, Inh*
ICC-101	New Delhi	S, R
ICC-102	New Delhi	S, R
ICC-103	New Delhi	R, R
ICC-104	New Delhi	R, R
ICC-105	New Delhi	R, R
ICC-107	New Delhi	S, S
ICC-109	New Delhi	S, R
ICC-114	New Delhi	S, R
ICC-1141	Jammu	S, S
ICC-124	New Delhi	S, R
ICC-128	New Delhi	R, R
ICC-132	Ahmedabad	S, S
ICC-133	Ahmedabad	R, R
ICC-137	Ahmedabad	S, R
ICC-138	Ahmedabad	S, S
ICC-144	Shimla	S, R
ICC-155	Chandigarh	S, R
ICC-161	Chandigarh	S, S
ICC-173	Chandigarh	S, S
ICC-174	Chandigarh	S, S
ICC-19	New Delhi	R,R
ICC-208	New Delhi	S, S
ICC-211	New Delhi	S, S
ICC-212	New Delhi	S, S
ICC-214	New Delhi	S, R
ICC-217	New Delhi	R, R
ICC-219	New Delhi	S, S
ICC-223	New Delhi	S, S
ICC-23	New Delhi	R, R
ICC-244	New Delhi	R, R
ICC-247	Chandigarh	R, R
ICC-248	Chandigarh	S, S
ICC-251	Chandigarh	S, S
ICC-254	Chandigarh	R, R
ICC-257	Chandigarh	R, S
ICC-275	New Delhi	R, S
ICC-286	New Delhi	R, R
ICC-327	New Delhi	R, R
ICC-33	Ahmedabad	R, R
ICC-332	Jaipur	R, R
ICC-37	Ahmedabad	R, R
ICC-399	Bangalore	R, S
ICC-95	Bangalore	S, S
ICC-96	Bangalore	R, R
ICC-98	New Delhi	S, S

*S, susceptible; R, resistant.

### TbD1 Analysis

The presence of TbD1 was analyzed by PCR (*28*). Briefly, 2 PCR assays were performed per isolate tested, by using either primers complementary to the sequences flanking the deleted region or primers complementary to the internal sequences. For the isolates that did (TbD1+) or did not (TbD1–) contain the TbD1 region, an amplicon was obtained only with internal primers or only with flanking primers, respectively.

### Single Nucleotide Polymorphism Analysis

To define the PGGs, the polymorphisms at the *katG* codon 463 and the *gyrA* codon 95 were determined by sequence analysis after PCR amplification with the same primers as in Sreevatsan et al. (*2*). The amplification products were sequenced by using an ABI 3700 DNA sequencer and the BigDye Terminator v3.1 Cycle sequencing kit (PE Applied Biosystems, Foster City, CA, USA).

### Spoligotyping

Spoligotyping was performed by using a commercial kit (Isogen Bioscience BV, Maarsen, the Netherlands) according to the previously described method (*29*). Reverse blotting analysis of spacer sequences in the DR region was performed by using a streptavidin-horseradish peroxidase–enhanced enzyme chemiluminescence assay (Amersham Pharmacia-Biotech, Roosendaal, the Netherlands).

### MIRU-VNTR Typing

The *M. tuberculosis* isolates were genotyped by PCR amplification of the 12 MIRU-VNTR loci (*18*) and 9 additional VNTR loci (*23**–**25*) by using an automated technique as described (*19*). The set of loci thus included (alternative designation in parentheses) MIRU-VNTR loci 2, 4 (ETR-D), 10, 16, 20, 23, 24, 26, 27, 31 (ETR-E), 39 and 40; and VNTR loci 424, 577 (ETR-C), 1895 (QUB-1895), 2347, 2401, 2461 (ETR-B), 3171, 3690, and 4156 (QUB-4156). The primers against the MIRU-VNTR flanking regions were the same as described (*18*), except that hex labeling was replaced by Vic labeling. The primers corresponding to the 9 additional VNTR loci and the conditions for their amplification by multiplex PCR are described in Table 3. The PCR fragments were sized and the various VNTR alleles were assigned after electrophoresis on a 96-well ABI 377 sequencer with customized GeneScan and Genotyper software packages (PE Applied Biosystems), as described (*19*) and on the basis of data described in Table 3. Tables used for MIRU-VNTR allele scoring are available at http://www.ibl.fr/mirus/mirus.html.

**Table 3:** Conditions for multiplex PCRs of 9 VNTR loci*

Multiplex	Locus	Conventional designation†	VNTR length (bp)	MgCl_2_ (mmol)	PCR primer pairs (5´ to 3´, with labeling indicated)
Mix E	46	VNTR 2347	57	1.5	GCCAGCCGCCGTGCATAAACCT (Fam) AGCCACCCGGTGTGCCTTGTATGAC
	48	VNTR 2461	57		ATGGCCACCCGATACCGCTTCAGT (Vic) CGACGGGCCATCTTGGATCAGCTAC
	49	VNTR 3171	54		GGTGCGCACCTGCTCCAGATAA (Ned) GGCTCTCATTGCTGGAGGGTTGTAC
Mix F	42	VNTR 0424	51	1.5	CTTGGCCGGCATCAAGCGCATTATT GGCAGCAGAGCCCGGGATTCTTC (Fam)
	43	VNTR 0577	58		CGAGAGTGGCAGTGGCGGTTATCT (Vic) AATGACTTGAACGCGCAAATTGTGA
	44	VNTR 1895	57		GTGAGCAGGCCCAGCAGACT (Ned) CCACGAAATGTTCAAACACCTCAAT
Mix G	47	VNTR 2401	58	3.0	CTTGAAGCCCCGGTCTCATCTGT (Fam) ACTTGAACCCCCACGCCCATTAGTA
	52	VNTR 3690	58		CGGTGGAGGCGATGAACGTCTTC (Vic) TAGAGCGGCACGGGGGAAAGCTTAG
	53	VNTR 4156	59		TGACCACGGATTGCTCTAGT GCCGGCGTCCATGTT (Ned)

*VNTR, variable-number tandem repeats. †VNTR 0577, 2461, 4156, 1895 correspond to ETRC, ETRB, QUB 4156 and 1895, respectively (*23**,**24*). The conditions for PCR amplification were the same as in (*30*).

### Analysis of Genetic Relationships

MIRU-VNTR profiles and spoligotypes were computed as character data into the Bionumerics program (Bionumerics version 2.5, Applied Maths, Saint-Martens-Latem, Belgium). MIRU-VNTR profiles were compared to each other by using the neighbor-joining algorithm. For spoligotypes, the Jaccard index was calculated to allow for the construction of a dendrogram by using the unweighted pair-group method with arithmetic averages. The spoligotypes were compared to fingerprints in an international database (*31*) that contained fingerprints from 13,008 isolates at the time of the consultation (June 2004). The genetic relationships between the isolates based on the MIRU-VNTR types were assessed by matching the spoligotypes with TbD1, and SNP analyses were carried out on a selected set of isolates representative of the different spoligotypes in each of the 3 predicted PGG1 groups and in the predicted PGG2/3 groups.

## Results

### MIRU-VNTR and Spoligotype Analysis

Nine of the 100 isolates of the study collection displayed 2 alleles in several independent MIRU-VNTR loci among the 21 tested, which suggested mixed DNA populations. These mixed populations could have originated from laboratory cross-contaminations or from mixed infections. Therefore, they were excluded from further analysis.

**Figure 1:**
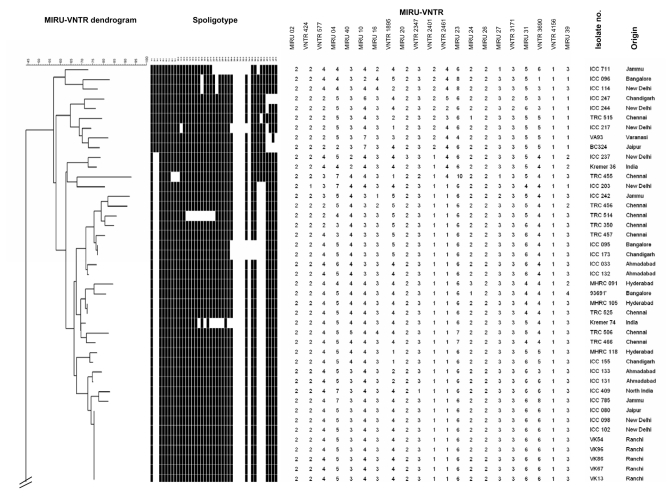
Genetic relationships of Indian Mycobacterium tuberculosis isolates. The dendrogram based on mycobacterial interspersed repetitive units–variable-number tandem repeats (MIRU-VNTR) genotypes, generated by using the neighbor-joining algorithm, was rooted with M. canettii, the most divergent member of the M. tuberculosis complex. Corresponding spoligotypes of the TbD1+/EAI isolates, additional genogroups, principal genetic group (PGG), and M. tuberculosis–specific deletion region 1 (TbD1) status are indicated. Indian isolates included in the collection of Kremer et al. (*16*) were included as references. LAM, Latin American–Mediterranean; CAS, Central Asian; EAI, East Africa–Indian.

The remaining 91 isolates showed highly diverse MIRU-VNTR genotypes (Figure 1). Seventy-eight distinct genotypes were detected in this collection, including 6 cluster patterns and 72 unique patterns. The largest MIRU-VNTR cluster included 8 isolates, 5 of which originated in Ranchi. Another cluster included 3 isolates from New Delhi, while the remaining 4 clusters contained 2 isolates each (1 with 2 isolates from Delhi; the others included isolates from Jammu and Chandigarh, from Hyderabad and Chennai, and from Bangalore and Chandigarh). Information about possible links between patients with clustered isolates was not available. The number of different spoligotypes (36 distinct spoligotypes, including 11 cluster patterns and 25 unique patterns) was lower than that of the MIRU-VNTR types, which was consistent with previous comparisons between spoligotyping and MIRU-VNTR systems based on 12 loci (*19**–**21*). None of the MIRU-VNTR clusters was split by spoligotyping, while of the 11 spoligotype clusters, 9 were split by MIRU-VNTR typing.

The genetic relationships between the isolates based on the MIRU-VNTR types by using the neighbor-joining algorithm are displayed in Figure 1. This dendrogram indicates 3 main genotype groups. The identity of these groups was inferred by comparison with genetically well-characterized isolates from a worldwide collection (*16*), typed by using the same 21 MIRU-VNTR loci (19; P. Supply et al., unpub. data). These groups are thereby predicted to correspond to the TbD1+ ancestral lineage (41 isolates, 45% of the total isolates), the recently described Delhi or Central Asian (CAS) genogroup (24 isolates, 26%), and the Beijing genogroup (9 isolates, 10%), respectively. These 3 groups belong to PGG1. The remaining isolates (17 isolates, 19%) are predicted to belong to PGG2 or PGG3 genogroups.

### Congruence of Groupings Between Markers

In accordance with the MIRU-VNTR typing results and the absence or presence of DR spacers 33–36 (*26*), *katG* and *gyrA* sequence analyses identified all tested representatives from Delhi and the ancestral genogroups as PGG1, whereas 14 tested isolates were assigned to PGG2 (Figure 1). One representative of PGG3, assumed to be the most recent group, was detected in this sample. The Beijing/W isolates were not tested for *katG* and *gyrA* polymorphism, since the fact that they all belong to PGG1 is well documented (*26*).

In agreement with Brosch et al. (*28*), we found the TbD1 region in all tested isolates from the predicted ancestral group but not in all tested Beijing/W, PGG2, and PGG3 isolates (Figure 1). We also found that all the tested Delhi isolates lacked TbD1.

Already known spoligotype signatures (*16**,**26**–**28**,**31**,**32*), and a few new variants, were found within the 4 groups defined by MIRU-VNTR analysis (Figure 1 and Table 4). The TbD1+ isolates were characterized by the absence of spacers 29 to 32 and 34, and (except in 4 cases) by the presence of spacer 33. Most isolates (35 of 43, taking into account 2 Indian isolates from the collection of Kremer et al. [16]) also lacked spacers 2 and 3. Based on these results, three fourths of the TbD1+ isolates were included in the spoligotype EAI3 class (*33*), while 1 isolate belonged to the EAI1 class. The remaining TbD1+ isolates represented new EAI variants. EAI classes 2, 4, and 5 were not found in this collection. Typically, the Beijing/W isolates only harbored spacers 35–43 (with spacers 39 and 40 missing in 1 case) (*5**,**32*). As described recently (*11*), the Delhi isolates shared the block of 9 final spacers (with some internal variation) with the Beijing/W strains but included 2 additional blocks among spacers 1–22. They specifically lacked spacers 4–7, and 23–34. The Delhi types are thus included in the CAS spoligotype family (*33*). Fourteen and 4 isolates out of 25 (taking into account 1 Indian isolate from the collection of Kremer et al. [16]) conformed to the 2 main spoligotype prototypes, CAS1 and CAS2, respectively (*33*).

**Table 4:** Specific spoligotype signatures observed in this study*

PGG	TbD1	Class	Spacers in DR region	
			Absent	Present
1	+	EAI1	29–32, 34, 40	All others
1	+	EAI3	2–3, 29–32, 34, 37–39	All others
1	+	EAIx	2–3, 29–32, 34	Most others
1	–	CAS1	4–7, 23–34	All others
1	–	CAS2	4–10, 23–34	All others
1	–	CASx	4–7 or 4–10, 23–34	Most others
1	–	Beijing	1–34	Most others
2–3	–	LAM, X, T	33–36	Most others

*DR, direct repeat; PGG, principal genetic grouping; TbD1, *M. tuberculosis*–specific deletion region 1; EAI, East African–Indian; CAS, Central Asian; LAM, Latin American–Mediterranean.

As expected, the prototypes of the Latin American–Mediterranean (LAM, 3 cases), X (1 case), and T (8 cases) spoligotype families were detected among the isolates of the PGG2/3group. The single PGG3 isolate (ICC399) had a T spoligotype, which includes both PGG2 and PGG3 strains (*26**,**33*). Highly similar groupings of the isolates were observed when a dendrogram was built based on spoligotypes alone or on a combination of VNTR and spoligotypes, although the resolution was lower when spoligotyping was used alone (data not shown).

### Comparison of Spoligotypes with an International Database

Table 5 shows the results from a comparison of the Indian spoligotypes with SpolDB3.0, a database containing data from >13,000 *M. tuberculosis* complex isolates obtained worldwide (*31*). Of 36 different spoligotypes found in the Indian strains, 15 (41.7%) were not present in SpolDB3.0. Most (11, 73%) of these new spoligotypes correspond to PGG1 isolates. Conversely, only 1 Indian isolate had the second most frequent spoligotype worldwide, S53. These observations reflect the current underrepresentation of strains from India in SpolDB3.0 (n = 44).

**Table 5:** Major spoligotypes in India*†

Spoligotype	India, no. (%)	SpolDB3.0, no. (%)	Geographic distribution‡
S11§	27 (28.7)	121 (1.03)	ASI, EUR, OCE, NAM, CAM, SAM
S26§	14 (14.9)	102 (0,87)	ASI, EUR, OCE, NAM, CAM
S1§	8 (8.5)	1,282 (10.95)	Ubiquitous
S52§	4 (4.3)	163 (1.39)	Ubiquitous
S288§	4 (4.3)	6 (0.051)	ASI, EUR, OCE, NAM
S138§	2 (2.1)	23 (0.20)	EUR, NAM, CAM
S342§	2 (2.1)	3 (0.026)	NAM
S357§	2 (2.1)	8 (0.068)	ASI, EUR, NAM
S361§	2 (2.1)	3 (0.026)	ASI, EUR
ICC09¶	2 (2.1)	–	India
ICC114¶	2 (2.1)	–	India

*n = 94. †Three Indian isolates from the collection of Kremer et al. (*16*) were included as references. Only spoligotypes shared by >2 isolates are shown. ‡Ubiquitous, spoligotype present in all 7 geographic areas; AFR, Africa; ASI, Asia; EUR, Europe; OCE, Oceania; SAM, South America; CAM, Central America; NAM, North America. §Designation of the spoligotype in SpolDB3.0. ¶New spoligotype not included in SpolDB3.0.

## Discussion

This report describes the diversity of *M. tuberculosis* strains obtained from patients in various regions in India, relying on a conveniently available set of isolates collected between 1997 and 2002. While these data are not representative of all TB patients in those regions and lack information regarding clinical characteristics, they provide valuable first insights into the diversity of circulating *M. tuberculosis* strains in this country. The excellent congruence observed between the 4 independent sets of genetic markers used here lends strong support to the assignment of different prevalent lineages. This congruence is consistent with the clonal population structure of *M. tuberculosis* (*2**–**4*) and reflects the respective informative values of the markers used. In particular, the results show that the use of a large set of VNTR loci simultaneously allows for both reliable identification of genogroups and high-resolution analysis of intralineage diversity, without the limitations that apply to IS*6110* fingerprinting or other typing methods used in the few previous molecular studies on Indian isolates. Within the framework of the current evolutionary scenario of *M. tuberculosis*, which proposes phylogenies based on PGGs and genomic deletion analyses (e.g., TbD1) (*2**,**3**,**28*), we found a striking prevalence of ancestral genotypes (TbD1+) and the concurrent poor representation of the most recent lineages in this Indian collection (PGG2 and especially PGG3). This finding contrasts with the situation in other regions of the world, such as Europe and North and South America, where PGG2 and PGG3 constitute most of the *M. tuberculosis* strains (*31*).

Ancestral isolates of *M. tuberculosis* are characterized by the presence of the TbD1 region, which has been recently identified as an evolutionary landmark in the genome of this species. This region was detected initially in a few *M. tuberculosis* strains belonging to PGG1, as well as in *M. canettii, M. bovis, M. africanum*, and *M. microti*, whereas this region was shown to be absent in all PGG2 and PGG3 strains as well as in the other PGG1 strains tested (*28*). The grouping of all the tested TbD1+ isolates by MIRU-VNTR typing and spoligotyping (16,19, this study) support their assignment to a single lineage (*28*), the East African–Indian lineage (*27*). Consistently, all tested representatives of known modern *M. tuberculosis* genotype families were TbD1–. A similar systematic association has recently been observed in strains from Singapore (*34*) and from Bangladesh (*35*), which supports the notion that the deletion of TbD1 occurred as a single evolutionary event in a common ancestor rather than on independent multiple occasions (*28*).

In this study, all isolates that contain >2 repeats in MIRU-VNTR locus 24 belong to the ancestral (TbD1+ group, and all but 2 isolates containing 1 repeat unit in locus 24 belong to the modern (TbD1–) groups. This correlation, also seen in previous studies on isolates from Singapore (*34*) and Bangladesh (*35*), indicates that this locus alone is highly informative in the identification of ancestral and modern *M. tuberculosis* strains.

**Figure 2:**
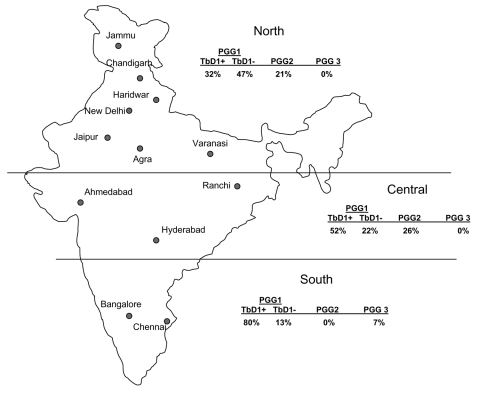
Geographic distribution of Mycobacterium. tuberculosis genotypes in northern, central, and southern India. PGG, principal genetic group; TbD1, M. tuberculosis–specific deletion region 1.

The few previously identified TbD1+ strains were isolated from patients from East Africa and South Asia. These strains have low copy numbers of IS*6110* (*16**,**28*) and belong to cluster I within PGG1 (*3*). This lineage is distinct from IS*6110* low-copy-number strains in PGG2 (*3*), which was isolated from patients in English-speaking countries (*33*). IS*6110* low- copy-number strains are prevalent with variable proportions among patients from several countries in Southeast Asia (*36*), and the analysis of the available spoligotype data suggests that most of them belong to the TbD1+/EAI lineage (*26**,**31**,**37*). Frequencies of TbD1+/EAI isolates have recently been reported to range from 25% to 50% in Bangladesh (*35**,**36*) and Singapore (*34*). A frequency of 8% has been reported in a study that only used spoligotyping for genetic characterization of 105 isolates from the Delhi area (*15*). Until now, the highest prevalence of IS*6110* low-copy-number isolates (≈60%) has been observed in southern India (*8**,**9**,**38*). Consistent with these studies, we found that 80% of the samples obtained from the southern regions from India were TbD1+/EAI isolates, although such isolates were found in nearly all regions (Figure 2). Also consistent with our findings, most spoligotypes observed in an ongoing population-based study (>1,200 isolates) in the southern state of Tamil Nadu were of the EAI3 class (S. Narayanan et al., unpub. data), found to be predominant in our collection (Table 4 and Figure 1).

The prevalence of these low-copy-number strains in regions of such high endemicity has raised the question of the true extent of genetic variation beyond their restricted IS*6110* distribution (*9*). The MIRU-VNTR typing results obtained here indicate that the genetic diversity in the TbD1+/EAI lineage goes far beyond the commonly observed restricted spectrum of IS*6110* low copy-number fingerprints and of known spoligotypes (*31**,**36*). For instance, most isolates with identical spoligotype 11 of the predominant EAI3 class in this study were of different MIRU-VNTR types (Figure 1). Moreover, the EAI lineage contains 3 additional spoligo-prototypes (EAI2, 4, and 5) that were absent from the population studied here. In addition, at least 1 group of TbD1+/EAI strains recently isolated in Singapore had high-copy numbers (up to 15) of IS*6110* (*39*). Altogether these observations indicate that the TbD1+ strains constitute a highly diversified lineage, which is consistent with an ancestral phylogenetic position.

In addition to the TbD1+ isolates, 2 other major PGG1 families were well represented in this Indian collection. They were qualified as modern groups by their TbD1– status. The recently identified Delhi type (*11*), classified as the CAS group by spoligotyping (*33*), represented approximately one fourth of the total sample. This genogroup made up 60 (72%) of 83 isolates collected from male patients attending 1 hospital and a clinic of the Delhi region over a 1-year period (*11*) and 38 (36%) of 105 isolates collected from patients attending other health centers in Delhi (*15*). Although this genogroup is less dominant in this region, representing 5 (20%) of the 26 isolates from Delhi, the Delhi genogroup was well represented among the isolates from northern and central India as well. The second TbD1– PGG1 family detected in this study corresponds to the widespread Beijing/W family, which accounted for 10% of the total sample. Most (7 of 9) of these isolates were from Delhi, where they represented 30% of the isolates studied, in contrast to the 1% to 8% noted in other studies on isolates from Delhi or other Indian regions (*11**,**15**,**32*).

The predominance of *M. tuberculosis* ancestral strains and the relatively poor representation of the most recent lineages in this Indian collection lend support to the hypothesis that India is a relatively ancient endemic focus of TB (*28*). On the basis of these findings, we speculate that the Indian subcontinent was an early step of the worldwide expansion of the *M. tuberculosis* complex, subsequent to the recently proposed emergence of tubercle bacilli in eastern Africa millions of years ago (*40*). However, we acknowledge that, as our collection represents a minuscule fraction of the millions of TB cases in India, genotyping additional isolates from TB patients in this country will be necessary to determine if these initial observations hold true (as suggested by unpublished data from >1,200 isolates from southern Tamil Nadu) or substantially change for a larger fraction of reported cases. Nevertheless, we believe that our data constitute the most solid available foundation for future comparisons of these additional isolates and those obtained from patients in the rest of the world.
